# Experiences and lessons learned from two virtual, hands-on microbiome bioinformatics workshops

**DOI:** 10.1371/journal.pcbi.1009056

**Published:** 2021-06-24

**Authors:** Matthew R. Dillon, Evan Bolyen, Anja Adamov, Aeriel Belk, Emily Borsom, Zachary Burcham, Justine W. Debelius, Heather Deel, Alex Emmons, Mehrbod Estaki, Chloe Herman, Christopher R. Keefe, Jamie T. Morton, Renato R. M. Oliveira, Andrew Sanchez, Anthony Simard, Yoshiki Vázquez-Baeza, Michal Ziemski, Hazuki E. Miwa, Terry A. Kerere, Carline Coote, Richard Bonneau, Rob Knight, Guilherme Oliveira, Piraveen Gopalasingam, Benjamin D. Kaehler, Emily K. Cope, Jessica L. Metcalf, Michael S. Robeson II, Nicholas A. Bokulich, J. Gregory Caporaso

**Affiliations:** 1 Center for Applied Microbiome Science, Pathogen and Microbiome Institute, Northern Arizona University, Flagstaff, Arizona, United States of America; 2 Laboratory of Food Systems Biotechnology, Institute of Food, Nutrition, and Health, ETH Zürich, Zürich, Switzerland; 3 Department of Animal Sciences, Cell and Molecular Biology Special Academic Unit, Colorado State University, Fort Collins, Colorado, United States of America; 4 Centre for Translational Microbiome Research, Department of Microbiology, Tumor, and Cancer Biology, Karolinska Institutet, Stockholm, Sweden; 5 Department of Pediatrics, University of California San Diego, San Diego, California, United States of America; 6 Center for Computational Biology, Flatiron Institute, Simons Foundation, New York, New York, United States of America; 7 Environmental Genomics, Instituto Tecnológico Vale, Belém, Pará, Brazil; 8 Center for Microbiome Innovation, Jacobs School of Engineering, University of California San Diego, San Diego, California, United States of America; 9 Academic Programs, Foundation for Advanced Education in the Sciences at the National Institutes of Health, Bethesda, Maryland, United States of America; 10 European Molecular Biology Laboratory, European Bioinformatics Institute (EMBL-EBI), Hinxton, Cambridgeshire, United Kingdom; 11 School of Science, University of New South Wales, Canberra, Australia; 12 Department of Biomedical Informatics, College of Medicine, University of Arkansas for Medical Sciences, Little Rock, Arkansas, United States of America; University of Toronto, CANADA

## Abstract

In October of 2020, in response to the Coronavirus Disease 2019 (COVID-19) pandemic, our team hosted our first fully online workshop teaching the QIIME 2 microbiome bioinformatics platform. We had 75 enrolled participants who joined from at least 25 different countries on 6 continents, and we had 22 instructors on 4 continents. In the 5-day workshop, participants worked hands-on with a cloud-based shared compute cluster that we deployed for this course. The event was well received, and participants provided feedback and suggestions in a postworkshop questionnaire. In January of 2021, we followed this workshop with a second fully online workshop, incorporating lessons from the first. Here, we present details on the technology and protocols that we used to run these workshops, focusing on the first workshop and then introducing changes made for the second workshop. We discuss what worked well, what didn’t work well, and what we plan to do differently in future workshops.

## Introduction

QIIME 2 (https://qiime2.org) is a widely used microbiome bioinformatics platform, with users around the world and working across all areas of microbiome research. Since its initial conception, the developers and others have taught hands-on workshops around the world in a wide variety of formats. These have sometimes been held at universities or research institutions for local teams of microbiome researchers; sometimes timed with microbiome-focused conferences as either official conference events or unofficial sessions before or after a conference such as the Soil Science Society of America (November 2, 2014) or the International Society for Microbial Ecology meetings (August 18, 2012 and August 30, 2014); and sometimes held in collaboration with nonprofit educational organizations such as the Foundation for Advanced Education in the Sciences (FAES) at the National Institutes of Health (NIH; February 22 to 23, 2018, December 12 to 14, 2018, and January 8 to 10, 2020). Workshop costs, such as instructor travel and cloud computing expenses, are often covered by the hosting institution or by the participants through a registration fee.

### Components of recent in-person workshops

Our recent in-person workshops are composed of different components (bolded in the following text) that we hoped to reproduce in an online event. The schedule of our most recent in-person workshop is presented in S1 Fig. **Lectures** covering basic theory on QIIME 2 [1], microbiome research, and bioinformatics are a large component. These typically cover topics such as approaches for sequence quality control, diversity metrics, taxonomic assignment methods, differential abundance testing, and QIIME 2’s semantic type and data provenance tracking systems.

Interspersed with our lectures, we have **hands-on tutorial sessions** where we guide participants through running QIIME 2 on a single tutorial data set that is used throughout the workshop. These tutorial data are derived from an actual microbiome study but filtered to a small fraction (around 10%) of the full data set to enable quick analysis. We deploy a shared compute cluster on Amazon Web Services (AWS) for the workshop, and all participants are given their own login credentials on this server. After a topic is introduced in a lecture, we guide participants through applying that new knowledge and interpreting the results. For example, in a lecture on beta diversity metrics, we cover unweighted UniFrac [2], how it is related to other diversity metrics, and how it is computed using an example simple enough to be computed with pencil and paper. We then have participants connect to the workshop cluster, and we guide them through using QIIME 2 to compute unweighted UniFrac on the tutorial data set and generate statistical and visual summaries of the results. They view the results that they generated using QIIME 2 View (https://view.qiime2.org), and we discuss the interpretation of those results as a group.

In addition to the lectures and hands-on work, we host dedicated **question and answer (Q&A) sessions** on general topics in microbiome research or QIIME 2. These are often the sessions of our workshops that participants report as the most valuable. To expand on the success of the Q&A sessions, we have recently begun to facilitate “**watercooler chats**,” where participants and instructors convene in small groups during coffee and lunch breaks to discuss topics of common interest that might not be relevant to all participants. An example of such a topic might be protocols for analyzing data generated with a new sequencing technology. Finally, in our longer workshops, we sometimes schedule a **poster session**, where attendees can present their own work, and a day of **parallel sessions** on topics of more specialized interest, such as analysis of fungal communities or developing QIIME 2 plug-ins. Parallel sessions allow participants to pick a path that suits their needs.

### Initial workshop plan and modifications for COVID-19

In 2019, the Caporaso Lab at Northern Arizona University received a grant from the Chan Zuckerberg Initiative (CZI), which included support for hosting a co-convened user and developer workshop in Latin America in collaboration with CABANA (https://www.cabana.online/), a project focused on increasing bioinformatics capacity in Latin America. We planned to test a new model for our workshops that would serve in part to foster interactions between the QIIME 2 user and developer communities. We planned to run a 5-day workshop where all participants would be invited to attend for all 5 days. Days 1 and 2 would be targeted toward users of QIIME 2, following the schedule of our successful 2-day QIIME 2 workshops. Days 4 and 5 would be targeted toward software developers and would cover topics such as plug-in and interface development and software testing. Day 3 would bring these communities together with a poster session, small group projects such as data analysis sprints, and lectures on topics of general interest, such as teaching QIIME 2 (many workshop participants subsequently teach informal workshops at their home institutions). We expected that some participants would not attend for the full week, but we planned to only accept participants who could be present for at least 3 of the 5 days to ensure full participation on Day 3.

This plan was disrupted by the Coronavirus Disease 2019 (COVID-19) pandemic, and, in March of 2020, we worked with CZI to reenvision our workshop as an online event. We had never taught an online QIIME 2 workshop before, but we had received many requests for one. We saw that this could still be viewed as an opportunity to develop a new model for our workshops that would let us reach a more diverse audience, such as individuals who couldn’t travel to a workshop due to financial or family constraints, and to host a more sustainable event by reducing its carbon footprint. These are widely recognized benefits of virtual rather than in-person events [3–5]. Since teaching an online workshop was new for us, we chose to teach this as a user-focused workshop (as opposed to our original plan of integrating user and developer workshops) to avoid making too many changes at once. We continued our plan to host a 5-day workshop in collaboration with CABANA. We decided to offer 75 seats for students to enroll and participate interactively in the workshop. We additionally planned to stream the workshop publicly on YouTube where other students could view the workshop in its entirety but not participate interactively. For clarity, we use the term **participants** to refer to the students who enrolled in the workshop and could participate interactively in the workshop and the term **viewers** to refer to the students who watched the livestream but were not enrolled, so couldn’t participate interactively. These terms and others are defined in Table 1.

**Table 1:** Workshop Role and Definition.

Participant	A student in the workshop who has full access to the event (including workshop server and Slack access). These are typically the paying attendees of the workshop.
Viewer	A student in the workshop who has view-only access through the YouTube stream. These individuals are attending for free and cannot access the workshop server or Slack, but can follow along with the workshop on their own deployment of QIIME 2.
Instructor	A teacher in the workshop. These individuals have contributed in one or more of the following ways: prerecording lectures or hands-on tutorial content, providing support to participants through Slack, joining live broadcast Q&A sessions, or assisting with the technical aspects of workshop delivery.
Technical leader	The instructor who is managing the broadcast software: queuing up prerecorded videos, transitioning title screens and overlays, and ensuring that the Skype-based speakers are connected to the broadcast.
Triage leader	The instructor who is leading moderation of the Slack workspace, including directing questions to other instructors or to queues to be addressed during live Q&A sessions.
Broadcast leader	The instructor who broadcasts live throughout the day, serving as a master of ceremonies. This instructor provides opening and closing notes at the end of each day, appears between all prerecorded videos to discuss upcoming events, breaks, etc., and acts as an “interviewer” during Q&A sessions.
Control room team	The technical leader, the triage leader, and the broadcast leader.
Workshop host	The organization that is providing the funding for the workshop event or who has otherwise coordinated the event and provided logistical and administrative support such as registration management, mediating student communications, evaluation development, and data analysis.

Q&A, question and answer.

## Results

### Student recruiting and registration

The CABANA team recruited 25 participants from 7 different countries in Latin America. These participants were selected considering a balance in gender, geographical location, minority groups participation, and how relevant the workshop would be to their work. The registration fee was US$50.

In addition to the CABANA cohort, we opened up 50 additional seats to the public. We charged a US$50 fee to participate in the workshop. Given the global target audience of the workshop, we recognized that US$50 might not be affordable to many people who may have been interested in attending. We therefore offered a purchasing power discount: Prospective participants could email to suggest an accessible price for the workshop, and we issued discount codes that those individuals could use when registering for the workshop. In order to not put any additional burden on these requestors, we did not solicit supporting material to evaluate their request. Instead, this was a “no questions asked” approach. We had approximately 140 total requests for purchasing power discounts. All discount requests were honored at the rate requested (including completely waiving the registration fee). Moreover, 15% of our 50-person public enrollment cohort of workshop participants enrolled with purchasing power discounts. We were inspired to adopt this model by The Pragmatic Studio (https://pragmaticstudio.com/about). Other groups have also used this approach to enable global participation in online bioinformatics events [4].

The US$50 fee for a 5-day workshop was considerably lower than a typical QIIME 2 workshop fee. Grant funding for this event enabled this, as we did not need to raise funds to cover our computing or personnel expenses. Because this was our first online event, we were also more comfortable doing a lower-cost event, acknowledging that our students were a test cohort for a new approach to teaching QIIME 2. We still felt that it was important to charge for attendance, to ensure that students who registered would be invested enough to show up for the workshop (a no-show at the workshop would have meant that an available seat was empty).

We received overwhelming demand for the workshop. We had initially planned to space registration out across time zones and a 1-week period to give prospective students the opportunity to enroll at a reasonable time of day in their location and on a day when they were available. Due to an error in our registration and payment system, however, all seats were made available at the first advertised time, and all available seats sold out in under 5 minutes. This is higher than the typical demand that we experience for our workshops and in line with other groups who have reported increased demand and reaching new audiences with virtual meetings [4,6].

**Fig 1:**
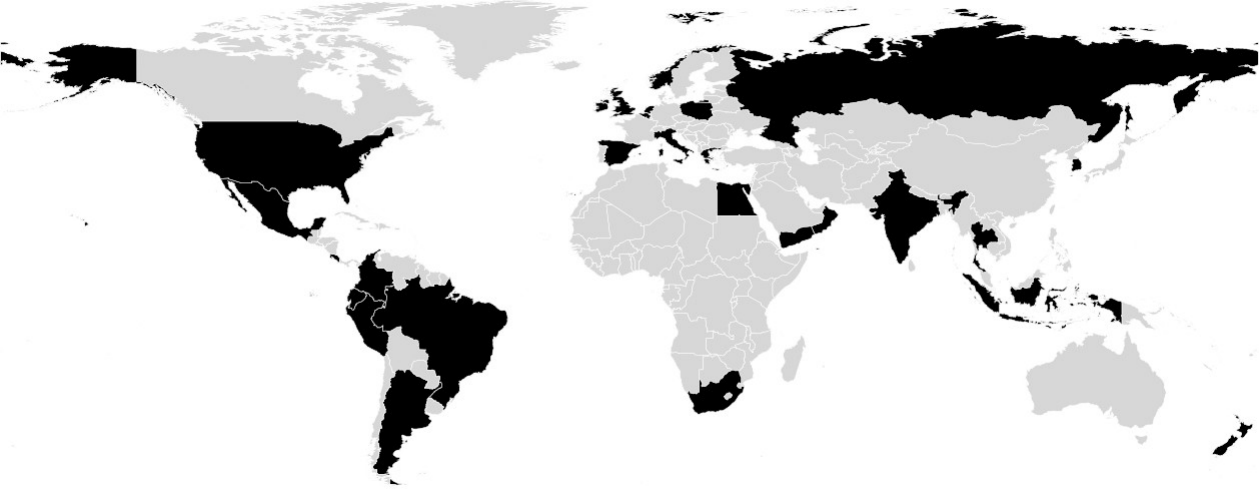
Map illustrating countries from which CZI/CABANA workshop participants attended (map base layer was plotted with data obtained from Natural Earth, https://www.naturalearthdata.com/). CZI, Chan Zuckerberg Initiative.

Enrolled participants joined the workshop from at least 25 countries (Fig 1). In addition to the participants who enrolled in the workshop, we streamed the workshop live on YouTube for free. Viewers of the livestream could see all of the presentations and Q&A sessions and could access the written tutorials that we worked through in the interactive portions of the workshop, but did not have access to the server or Slack workspace that were available to the students. This had 2 main implications. First, they could not ask questions of the workshop instructors (questions were asked on Slack, as detailed below). Second, they could not follow along with interactive components of the workshop unless they had their own deployment of QIIME 2 to work in. Thus, the participants paid for live access to the instruction team through Slack and for the use of our cloud-based workshop server. We set up spaces on the QIIME 2 Forum for viewers to discuss workshop content among themselves, and workshop instructors were reasonably successful in supporting workshop-related forum topics asynchronously while the CZI/CABANA workshop was in progress, although we prioritized supporting participants over viewers.

### Participant and instructor demographics

We performed a preworkshop questionnaire to compile information about our participants before we began teaching. A total of 71 out of 75 participants completed the preworkshop questionnaire, and we describe the background of the participants based on those results.

A total of 50% of our participants were graduate students, 20% were postdoctoral researchers, 18% were research staff, and 6% were faculty members. When asked to report their discipline or disciplines, 63% of participants reported Microbiology, 35% reported Agriculture or Environmental Sciences, 28% reported Bioinformatics, 23% reported Biomedical or Human Health Sciences, and smaller percentages of participants reported other disciplines including Chemistry, Planetary Sciences, and Education. This illustrates the reach of microbiome sciences and the associated bioinformatics tools across disciplines. Moreover, 73% of our participants reported using programming languages several times per year or more, but the majority reported never using databases such as SQL or Access (59.2%) or version control software (65%). A total of 65% reported using a command shell at least several times per year. Taken together, we interpret this as our cohort being relatively novice users of computing tools such as programming languages and command line software, and generally not comfortable with software engineering given the lack of experience with databases and revision control software. In addition, 56% of our participants reported using QIIME 2 at least several times per year, but less than 10% reported using QIIME 2 weekly or daily. Only 14% of our participants reported using QIIME 1 at least several times per year. QIIME 2 succeeded QIIME 1 on 1 January 2018. In general, we find that some of our workshop participants have experience with QIIME 1 and attend to update their skills.

We do not have historical questionnaire data to compare this cohort to previous cohorts. However, based on our experiences, the skills and background seem comparable to our in-person workshops. We plan to incorporate this questionnaire in all future QIIME 2 workshops, because the knowledge we gained about the participants was helpful for targeting discussions to their background during the workshop. The preworkshop questionnaire that we provided is available as S1 Text. It was derived from The Carpentries, where it is made available under the Creative Commons Attribution (CC-BY) license. The original materials are available at https://github.com/carpentries/assessment.

We reached out to instructors who had been involved in our recent workshops to recruit a team to co-teach our first online workshop. In addition to high demand from students, we had many instructors interested in co-teaching this workshop. A total of 22 instructors were involved in one or more of the following aspects of the workshop: prerecording lectures or hands-on tutorial content, providing support to participants through Slack, joining live broadcast Q&A sessions, or assisting with the technical aspects of workshop delivery. Our instructor team consisted of 1 undergraduate student, 9 graduate students, 3 postdoctoral scholars, 3 research staff, 4 assistant professors, and 2 associate professors.

We allowed instructors to select which topic or topics they would like to teach, based on a draft workshop schedule that we derived from recent workshops. Most novice QIIME 2 instructors were given first choice of topics, so they could select those they were most comfortable teaching.

## Materials and methods

### Technology stack

We purchased licenses of TechSmith Camtasia 2020 (https://www.techsmith.com/video-editor.html) for instructors, and this was the primary software used to record videos. Camtasia allows for simultaneous video and screen recording and provides many helpful features for video editing. For example, their Freeze Region visual effect allows a video editor to freeze a region of a screencast for a duration of this video, which enables editing out of pop-up notifications that may have occurred in the middle of a screen recording.

Camtasia 2020 only includes auto-captioning of videos for an extra fee, so we used other software to add captions. Our team experimented with both Otter.ai and YouTube Studio for auto-captioning. Both worked surprisingly well, but the auto-generated captions are of course not perfect, and considerable time is still needed for copyediting. Due to time constraints, copyedited captions were not included in the videos presented in the first workshop. Some were added for the second workshop, and copyedited captions are being added to all videos being released on the YouTube channel.

Instructors were given a deadline by which they needed to share their video recordings for review. We purchased a license for TechSmith Video Review (https://www.techsmith.com/video-review.html), which facilitates reviewing videos by allowing reviewers to link comments and annotations to frames in the video timeline (among other conveniences). We found these TechSmith tools to be very convenient for content creation, review, and editing, and the learning curve for basic usage was minimal.

During the workshop, the tools used to support our livestream were Open Broadcaster Software (OBS) Studio (https://obsproject.com), Skype, and YouTube. One instructor (the technical lead) ran OBS Studio, which fed our livestream to YouTube where it was broadcast publicly at a new URL for each day of the workshop. The technical lead maintained the daily schedule, which alternated between live broadcasts and prerecorded videos. Live broadcasts were conducted using Skype, in a Skype video conference that all instructors could join at any time. Skype was chosen over other video conferencing software (like Zoom) because it most easily integrated with OBS Studio through the NDI protocol (https://en.wikipedia.org/wiki/Network_Device_Interface), an industry-standard broadcast stream tool.

To facilitate hands-on work, we preloaded the tutorial data on the shared compute cluster. This server configuration was almost identical to the configuration we have used for our in-person workshops, except that some extra security precautions were taken since the workshop was livestreamed on YouTube, and, therefore, had a higher chance of being noticed by individuals who were not authorized to access the server. For this workshop, our cluster was composed of 13 m5.8xlarge machine instances (the compute nodes) and 1 m5.2xlarge machine instance (the login node). A total of 8 user accounts were assigned to each compute node. Participants are given their own login credentials to the system. We tend to include 1 or 2 extra compute nodes in these clusters so that if a compute node becomes inaccessible during the workshop, we can move participants to another one. This has happened on multiple occasions during our in-person workshops.

To connect to their compute node, all participants connect to the single login node of the cluster by SSH, which redirects them to the compute node they will work on for the workshop. This redirect is handled automatically without participant interaction. Participants perform their work in directories that are hosted over HTTP, so they can access the data they generate in their web browser. To view interactive QIIME 2 results, we have the participants copy URLs to the files they’ve generated from their web browser and paste the URL into QIIME 2 View, which allows them to interact with the results in their web browser.

Before the workshop, we have participants install Google Chrome and the Google Chrome Secure Shell App, which provides an SSH client that they can access through Google Chrome. This allows us to provide connection instructions that work across operating systems with a unified user interface, as our users may be working on macOS, Windows, or Linux. Participants sometimes ask if they can use their own SSH client. We always approve this, but we let them know that it may be harder for us to help them because we may not be familiar with their SSH client (in practice, we find that participants who are experienced enough to have a preferred SSH client often don’t need help navigating connection to the server). Using the Google Chrome Secure Shell App, QIIME 2 View, and the cluster web server’s index page for their data directory, participants can perform all hands-on steps of the workshop in their web browser. Participants were provided with a PDF “cheat sheet” that included graphical instructions for connecting to the server by SSH and accessing their data (example provided as S2 Fig). This sheet included connection details (hostname, username, and password) which they could copy and paste.

Throughout the week of our online workshop, we had a paid Slack workspace, which all workshop participants were given access to. We configured the Slack workspace with several private instructor-only channels and several channels that all participants had access to. Additionally, we created Slack channels for subgroups of participants which we referred to as “pod” channels, and each participant was preassigned to 1 pod channel. We hoped to use the pod channels to capture the “neighborhood” dynamic that tends to arise in our in-person workshops, where clusters of individuals sitting near one another begin helping each other debug issues or understand concepts. Slack was used to facilitate all contact between participants and instructors, and we used instant messaging, video, and audio calls to communicate with participants either individually or in groups as needs arose. Details on how we specifically used Slack are presented below.

### Instructor roles

In addition to delivering workshop content, 3 instructors had roles in workshop delivery: the technical leader, the broadcast leader, and the triage leader. These individuals formed the control room team. The technical leader was primarily responsible for running OBS Studio, and, thereby, controlling the livestream. The broadcast leader served as the master of ceremonies for the event, providing opening and closing remarks each day, reiterating the schedule, and serving as a consistent voice throughout the workshop. The triage leader was primarily responsible for monitoring Slack. This involved directing incoming questions for instructors to handle right away or to queues to be addressed during open Q&A sessions.

Due to the pandemic, the instructors were not generally colocated. Only the technical leader and the triage leader were colocated, both working on campus at Northern Arizona University, enabling the livestream to be broadcast over the university’s internet connection. These 2 instructors both received negative COVID-19 tests within the 48 hours preceding the workshop and worked in separate offices with windows opened in the same office suite to ensure appropriate social distancing. We chose to have 2 instructors working together even though, technically, only one was needed to manage OBS Studio, in case backup was needed. These instructors did get together for about 10 minutes in the same office, with the window and door opened, so the triage leader could be trained on OBS Studio. Masks were worn during this interaction, and social distancing was maintained.

### Workshop schedule

**Fig 2:**
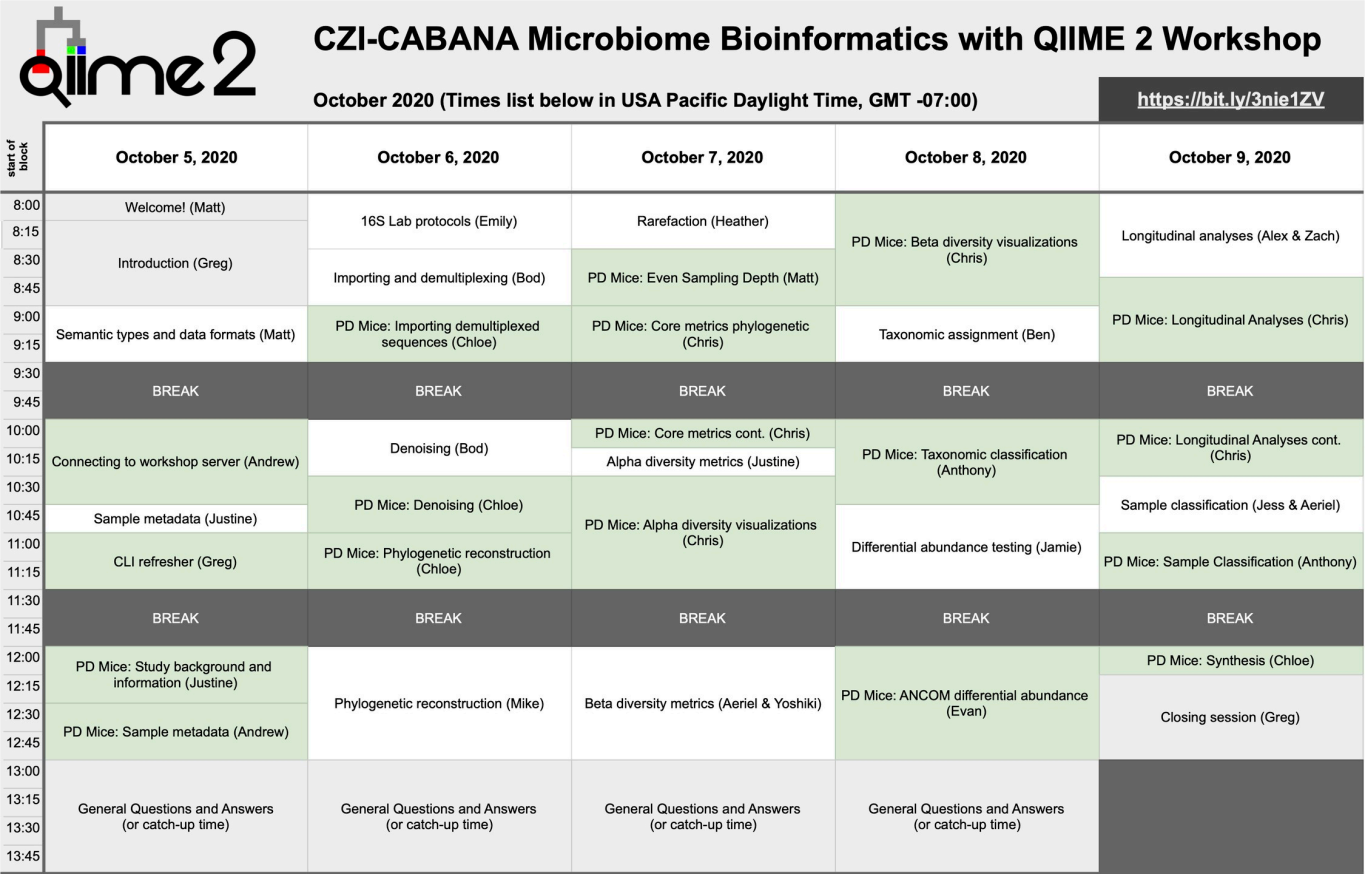
CZI/CABANA workshop schedule. CZI, Chan Zuckerberg Initiative.

The full workshop schedule is presented in Fig 2. A workshop day typically proceeded as follows. All times noted here are the United States of America Mountain Standard Time, corresponding to the time zone that the workshop was delivered from. The workshop ran from 8:00 to 14:00 on Days 1 to 4 and 8:00 to 13:00 on Day 5. At about 7:30, our livestream broadcast would begin, and we would share the link to the day’s broadcast on Slack (for participants) and on the QIIME 2 Forum (for viewers). A static slide would be presented on the livestream, indicating that the workshop would begin shortly. At this time, the instructor’s Skype video conference was initiated for the day. The control room team would join the Skype video conference, and other instructors might also join to say hello and to help with any last-minute tasks that needed to be accomplished for the day. At 8:00, the day’s broadcast would begin with the broadcast leader opening the session to describe the schedule for the day and to share any announcements. The livestream would then transition to the first prerecorded video for the day. Prerecorded videos ranged in length from 4 minutes to 64 minutes, with a median length of 22 minutes. Between each pair of prerecorded videos, the broadcast leader would join the livestream. Depending on the schedule, this time would sometimes be used to answer questions about the content that was just presented. All available instructors, ideally including the instructor whose prerecorded video was just streamed, would join the livestream through the instructor Skype to answer questions.

The day would proceed, presenting lecture content mixed with hands-on work. Two 30-minute breaks were included in the schedule: the first from 9:30 to 10:00 and the second from 11:30 to 12:00. We committed to sticking to our break times, sometimes pausing the livestream during a video. This is essential so participants and viewers can plan for their family, work, and other responsibilities during the workshop week. On the first 4 days of the workshop, the day would conclude with a 1-hour open Q&A session from 13:00 to 14:00. During this time, all available instructors would join the instructor Skype, and questions about any of the content presented or general questions about QIIME 2 or microbiome research were fielded by the instructors. If at any point during the Q&A sessions there were fewer questions, the broadcast leader would ask questions of the instructors currently on the call—for example, all instructors who joined these sessions were asked at some point during the week to describe the path they took to getting involved with microbiome research and QIIME 2. This allowed us to present the diverse career paths that lead to work in biomedical research and bioinformatics, which we hope is useful for encouraging participants and viewers from diverse backgrounds to pursue their interest in microbiome bioinformatics. At the end of each day’s Q&A session, the broadcast leader would conclude with any final announcements for the day. Within the hour following each day’s events, links to the prerecorded videos for the next day were shared with participants through Slack, so they could watch those in advance if that was helpful for their schedule the following day.

On the final day of the workshop, we concluded with a summary presentation and acknowledgments of all instructors who were involved in the workshop. All available instructors joined the livestream by chat to conclude and thank the participants and viewers for attending the workshop.

## Discussion

### Recreating in-person workshop online

The lecture component of our courses was relatively straightforward to recreate for an online workshop. We planned for specific topics that we wanted to cover in this workshop and reached out to a team of individuals who have been involved in previous QIIME 2 workshops and invited them to prerecord lectures on these topics. Prerecording lectures rather than presenting live is a popular option for virtual meetings [6]. It facilitates participation across time zones for both instructors and participants, reduces chances for technical hurdles during the workshop (e.g., if an instructor’s internet connection becomes unstable while presenting), and reduces the amount of work during the event so the hosts can focus their attention on serving the participants during that time. The prerecorded videos were made available to participants the day before they were to be covered in the workshop, allowing them to watch the videos when it was most convenient for them and at their own pace (enabling pausing of the video as needed to self-study or debug an interactive step). Then, during the live broadcast, we played back the same prerecorded videos for participants and viewers. In addition to providing flexibility for participants to view videos in advance if they had a conflict during a workshop day’s events, this gave participants the opportunity to watch the video before and during the workshop.

In our in-person workshops, the lines are often blurred between lectures and hands-on tutorial sessions. For example, an instructor might have the participants start a long-running command before they teach what it does so that it will complete by the time the lecture is completed. For the online workshop, we chose to separate lecture content and hands-on tutorial content into different prerecorded videos for a few reasons. First, this helped us to ensure continuity between hands-on sessions. Over the course of the workshop, the analyses that participants ran were built on previous steps of the analysis. So, if a file needs to exist at the beginning of one hands-on session, it needs to be generated—with the expected file path—in a previous section. Separating lecture and hands-on tutorial sessions kept the recordings shorter, making it quicker to rerecord tutorial sessions if errors were discovered during video review. Second, this approach allowed instructors who were recording lectures to focus on the content they wanted to teach and not the technical aspects of how participants would be using QIIME 2 during the workshop. Third, an auxiliary goal for our online workshop was to create video content that could be reused, and, in some cases, that could stand alone without the rest of the workshop content. Separating lecture content from tutorial content enabled this, since tutorial content wouldn’t necessarily make sense to someone watching a single video without the context of the other videos. Similarly, keeping lecture and tutorial content separate facilitates the reuse of lecture content in future workshops. While both types of video will undoubtedly need to be rerecorded in the future to keep pace with the field, we expect that tutorial content likely needs more frequent updating to support new versions of QIIME 2 (which are released quarterly). Finally, splitting lecture content and tutorial content provided opportunities for less-experienced instructors to get involved. One undergraduate student, 2 graduate students, and 1 junior research software engineer in the Caporaso Lab—none of whom had taught a QIIME 2 workshop before—taught the majority of the hands-on tutorial sessions for this workshop.

Q&A sessions are frequently noted as among the most valuable sessions by participants in our in-person workshops, so we put a lot of effort into trying to recreate that experience in our online workshop. In our in-person workshops, brief Q&A sessions are held after each lecture, and longer Q&A sessions are scheduled at the end of each day. We try to target general questions to the longer sessions and keep shorter sessions focused on content that was most recently presented. A frequent challenge that we encounter in the Q&A sessions at in-person workshops is keeping the discussion focused around topics that are likely to be of general interest, opposed to delving into topics that are relevant only to a single person’s analysis. We try to handle individualized questions through one-on-one discussions between an instructor and a participant, time permitting. If time is not available (e.g., a participant asks a very specific question at the end of the workshop), we direct participants to the QIIME 2 Forum.

**Fig 3:**
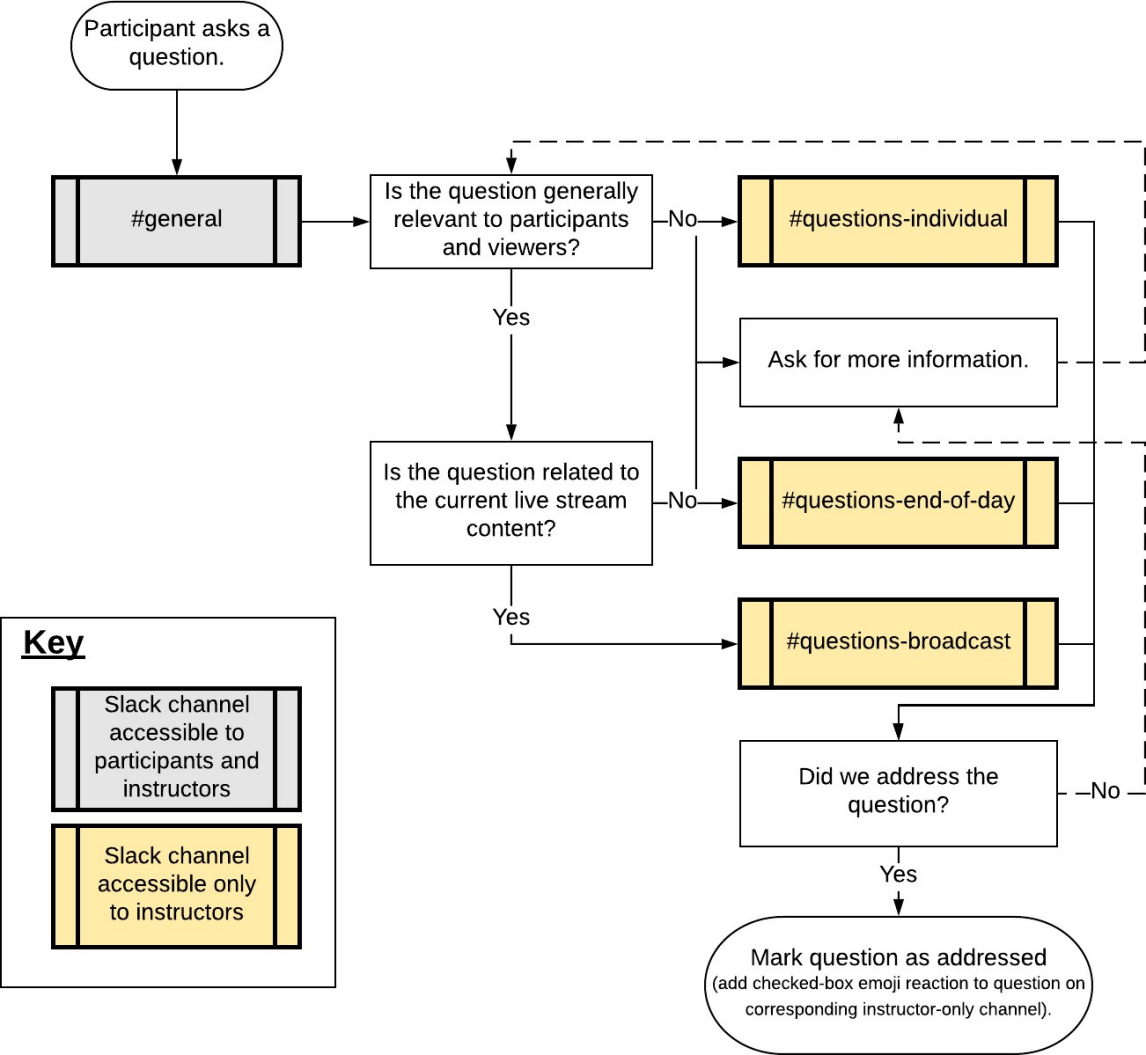
Triage protocol for questions from participants. All questions from participants were asked on Slack. Questions were triaged by the triage leader as they were asked.

In our online workshop, we handled user questions through Slack. A flowchart illustrating how these questions were triaged and addressed is presented in Fig 3. Questions were asked on the #general Slack channel, which all participants had access to. A moderator on the instruction team would forward these questions to 1 of 3 instructors-only channels: #questions-end-of-day, #questions-broadcast, or #questions-individual. The #questions-end-of-day channel was intended for more general questions that would be covered in the general Q&A session at the end of the day. When a participant asked a question on Slack that we redirected to this channel, we would reply to them inline to let them know we had logged the question and would bring it up later. The #questions-broadcast channel was intended for questions that should be addressed on the broadcast directly following a session because they were relevant to the most recent content. This channel also served as a broadcast-ready channel: A direct capture of the channel was overlaid into the live feed to show everyone watching the source of the question. If we ran out of time for one of these questions, we would move it to the #questions-end-of-day channel. The #questions-individual channel was intended for the questions that were very specific to a participant’s own project and not likely to be of general interest. Instructors monitored this channel and followed up directly with the participants to answer their questions on Slack (either in chat or in a Slack video call). At all times, one instructor was monitoring the #general channel to triage incoming questions. The other type of question that would come up during sessions was technical support requests, for example, when a participant needed help with a step in the tutorial because they observed an error or had a technical issue. The triaging instructor would forward these questions to the #questions-individual channel, and an instructor would follow up with participants in their pod channel. In general, we always tried to answer technical support and individual questions in pod channels so others with the same question could see the discussion. This helps others learn from the discussion and reduces the support burden on the instructor team. This system worked well. We found that it was easier to keep the Q&A sessions on target in this format than in in-person workshops, primarily because triage didn’t need to happen in real time in front of an audience.

We additionally set up 3 *#*watercooler-chat channels that were intended for small-group discussions (by chat or video) around a topic of interest. These ended up being widely used for introductions among the participants and were a good space for instructors and participants to chat about topics not directly related to the workshop content. For example, people shared where they were attending the workshop and pictures of their pets. Small-group discussions about workshop-relevant topics didn’t happen much in these channels, but they emerged as a way to get to know each other without the usual coffee breaks, lunches, and dinners that happen at our in-person workshops.

We did not host parallel sessions in this workshop, primarily because we didn’t have time to prepare the additional video content that would be required, and we didn’t want to attempt too many new things on this first online workshop. These could be achieved by running multiple video streams at dedicated times throughout the workshop, and we may revisit this idea for future online workshops.

### Outcomes

We performed a postworkshop survey of participants to evaluate the success of the workshop. A total of 41 participants (55% of those attending the event) responded to this survey. Because our surveys were performed on the last day of the workshop, it is possible that replies are biased by attendees who found the event valuable enough to stay until the end. We present survey responses in S1 Data and summarize our findings here.

Based on postworkshop survey responses, we consider this to have been an extremely successful workshop. A total of 98% of respondents reported that the workshop helped them learn what they most hoped to learn from participating in the workshop. Moreover, 93% agreed or strongly agreed that they could immediately apply what they learned at the workshop. In addition, 100% agreed or strongly agreed that they felt comfortable learning in the workshop environment. Furthermore, 93% agreed or strongly agreed that they were able to get clear answers to their questions from the instructors. Additionally, 95% strongly agree that the instructors were enthusiastic about the workshop. A total of 95% agreed or strongly agreed that they felt comfortable interacting with the instructors. Moreover, 98% agreed or strongly agreed that the instructors were knowledgeable about the material being taught. And, finally, 98% reported that they were likely or highly likely to recommend this workshop to a friend or colleague.

We additionally polled attendees on accessibility of the workshop and how much of the workshop they attended. A total of 93% of respondents reported no accessibility issues. Of the 7% who did report accessibility issues, the issues were related to their own internet connectivity problems or other work commitments distracting them from the workshop. Both of these are drawbacks to an online workshop that participants join from their own locations. Moreover, 78% of respondents reported being able to complete all of the interactive work, while 22% reported being able to complete some of the interactive work. No respondents reported not being able to complete any of the interactive work or not trying to complete the interactive work. This suggests that our AWS approach, paired with the Google Chrome Secure Shell client, was effective for allowing participants from around the world to simultaneously run QIIME 2 on the same infrastructure (something we were not certain of before the workshop). A total of 100% of the respondents reported attending all or some of the sessions each day, and none of the respondents reported attending none of the sessions on any day. (This question is particularly sensitive to bias, because if a participant didn’t attend the workshop on Day 5, they would have been more likely to miss our request to complete this survey. They would have been able to access the survey still, however, since the link and the request was shared on Slack in addition to being discussed during the workshop.) The percentage of participants who reported attending all sessions in a given day declined slightly over the course of the workshop: On Day 1, 95% reported attending all of the sessions; on Day 2, 95%; on Day 3, 90%; on Day 4, 73%; and on Day 5, 78%.

### Our second online workshop

In January of 2021, we presented a second online workshop. This workshop was hosted by the FAES at the NIH. FAES is a nonprofit organization that was created by NIH researchers to build a university-like environment at the NIH. FAES offers educational and training activities to the greater scientific community. This workshop followed a very similar approach as our first workshop, with a few modifications. In the remaining discussion, we refer to the first workshop as the CZI/CABANA workshop and the second workshop as the FAES workshop.

FAES offered a tiered pricing structure to accommodate individuals representing different industries where discounts were factored into the tiers (e.g., members of the NIH community and those affiliated with other US government, US military, or academic institutions can attend the workshop at a lower cost than those not affiliated with any of these groups). We had 57 participants and 24 instructors in this workshop, and the livestream for this event was not shared publicly. While this workshop was available for global participation, the majority of our attendees were from NIH and North American universities (approximately 85% of participants were from institutions in the USA or Canada).

The majority of the prerecorded content developed for the first workshop was reused in the second workshop. All live sessions in the first workshop were again performed as live sessions in this workshop. The daily schedules were largely the same as for the first workshop. We made a few changes to our technology and instructor roles for the second workshop.

First, instead of a paid Slack workspace for this workshop, we hosted our own Zulip (https://zulip.com/) server. This was a lower cost option because the pricing plan for Slack involves paying a fee per user. Administration and creation of user accounts was much quicker in Zulip than in Slack. Zulip supports bulk invitation using a CSV file, while Slack requires filling form fields for each user account. Zulip also has a command line interface for administration, which was a natural fit for the programmers on our team. This interface allowed us to quickly customize Zulip to our liking.

Zulip has a similar user interface to other chat platforms like Slack or Microsoft Teams, so even though many of the attendees told us they had not worked with Zulip before, they were able to become proficient relatively quickly. We hosted our Zulip server on a Digital Ocean droplet (2 GB/2 vCPUs/US$15 month) and provided a basic login and usage tutorial for users. Zulip uses a threaded discussion model where conversations are organized by theme in a Zulip “stream.” Individual discussions occur within a theme’s list of “topics,” which are chat threads created by users and instructors. This is different from the traditional “chat room” approach where all of a room or theme’s discussion occurs all at once, in a single location. We found that this model was not intuitive to many participants at first: We saw multiple parallel topics starting at once, which made supporting workshop participants slightly more difficult. Zulip does allow for moderator intervention in the form of moving and merging discussion topics, which helped manage the discussion (e.g., by allowing us to merge independent discussions of the same topic). After the first day of the workshop, we started preemptively creating new stream topics for each video/segment. We then sent notifications to all attendees asking them to post their questions about the current video inside the topic we created instead of creating their own. By the end of the second day, this approach appeared to work well for everyone. In future workshops using Zulip, we plan to dedicate more time in the beginning of the workshop providing guidance on how to use streams, topics, and notifications to reduce confusion.

The second change we made in this workshop was that we excluded the role of triage manager, opting instead for all instructors to assist with this. We felt that this was not as well organized, and, in the future, will add the triage manager back.

A total of 46 participants completed our preworkshop survey, which was identical to the preworkshop survey used for the CZI/CABANA workshop. Moreover, 24% of our participants were graduate students, 24% were postdoctoral researchers, 15% were faculty members, 7% were medical professionals, and 26% were research staff or government employees. In general, this suggests that our FAES workshop cohort were at later career stages than our CZI/CABANA workshop cohort. Furthermore, 72% of our participants reported using programming languages several times per year or more, but the majority reported never using databases such as SQL or Access (59%) or version control software (70%). In addition, 54% reported using a command shell at least several times per year. This cohort was thus very similar to our CZI/CABANA workshop participant cohort in terms of their experience with advanced computing tools. Also, 24% of our participants reported using QIIME 2 at least several times per year, and less than 5% reported using QIIME 2 weekly or daily. Only 7% of our participants reported using QIIME 1 at least several times per year. These findings suggest that our FAES cohort was generally less experienced with QIIME 1 or QIIME 2 than our CZI/CABANA cohort.

We performed a postworkshop survey of participants to evaluate the success of the workshop. All 57 participants responded to this survey, and based on these responses, we also consider this workshop to have been very successful. We present survey responses in S1 Data and summarize our findings here. A total of 96% of respondents reported that the workshop helped them learn what they most hoped to learn from participating in the workshop. Moreover, 77% agreed or strongly agreed that they could immediately apply what they learned at the workshop. Also, 91% agreed or strongly agreed that they felt comfortable learning in the workshop environment. Furthermore, 93% agreed or strongly agreed that they were able to get clear answers to their questions from the instructors. In addition, 100% agreed or strongly agreed that the instructors were enthusiastic about the workshop. A total of 98% agreed or strongly agreed that they felt comfortable interacting with the instructors. Moreover, 100% agreed or strongly agreed that the instructors were knowledgeable about the material being taught. And, finally, 93% reported that they were likely or highly likely to recommend this workshop to a friend or colleague.

We again polled attendees on accessibility of the workshop and how much of the workshop they attended. A total of 93% of respondents reported no accessibility issues. Of the 7% who did report accessibility issues, the issues were related to their own internet connectivity problems. Moreover, 91% of respondents reported being able to complete all of the interactive work, while the remaining 9% reported being able to complete some of the interactive work. No respondents reported not being able to complete any of the interactive work or not trying to complete the interactive work. In addition, 100% of the respondents reported attending all or some of the sessions each day, and none of the respondents reported attending none of the sessions on any day. The percentage of participants who reported attending all sessions in a given day varied slightly over the course of the workshop and was even higher than for the CZI/CABANA workshop: On Day 1, 100% reported attending all of the sessions; on Day 2, 98%; on Day 3, 93%; on Day 4, 88%; and on Day 5, 96%. This suggests to us that participants found considerable value in the workshop throughout the week.

### Lessons learned and improvements for future online workshops

We asked open-ended questions of participants at both workshops to compile information on what they liked, what they didn’t like, their suggestions for improvements, and what they found to be the most useful parts of the workshop. The features that respondents liked included the degree of organization, including our adherence to the schedule and providing regular breaks. Multiple respondents reported they liked having access to the prerecorded lectures before a given day’s session, sometimes noting that it helped them to watch the videos ahead of time. The Q&A sessions were very popular, and a lot of respondents reported that Slack/Zulip worked well for this. We also received positive feedback on the quality of the lectures and the choice of content. Some individuals noted that they prefer the online format and that instant communication through Slack/Zulip made it feel as though “we were in the same room.” One individual noted that “Everything was perfect”—we’re glad it appeared that way!

When polled on what aspects of the workshop they didn’t like, or which didn’t work well for them, the most common comment was that some of the lectures moved too fast at times (and some participants in the CZI/CABANA cohort reported that our accents posed a challenge to following the material). We did receive some consistent feedback on which specific lectures moved too quickly and which moved at a good pace, so that gives us guidance on which videos need attention. One respondent noted that the 30-minute breaks were long for individuals who were attending the workshop during the night in their local time. This is clearly a drawback of hosting a workshop for individuals from all over the world at the same time (other individuals noted that the breaks were well timed and important for allowing them to attend the workshop—this is likely reflective of how well aligned a participant’s time zone is with the workshop schedule). Several respondents noted that they would have preferred to have QIIME 2 installed on their own computers, as they left the workshop confused on how to run QIIME 2 when they no longer had access to the workshop server. Several respondents also mentioned that they missed the opportunity to gather in person for the workshop, including the opportunity to meet the instructors and other participants in person: a sentiment that we empathize with a year into the pandemic.

We received many excellent suggestions on how to improve future offerings. Several individuals reported that the workshop moved too fast, while others indicated that they wish we covered some more advanced usage of QIIME 2. This suggests to us that we should begin offering 2 tiers of workshop: a basic and advanced workshop. Our basic workshop could potentially spend more time on getting started with QIIME 2, including installation clinics (which are popular in parallel sessions at our in-person workshops), while our advanced workshop could include more challenges focused around understanding command line documentation to construct and run commands that complete some analysis challenge and working with noisier data. It was also suggested that daily quizzes could be added to help participants gauge whether they mastered the concepts we most hoped to teach in a given day. Several participants suggested that we provide instructions ahead of the workshop to optimize their learning environment, including perhaps using dual displays and suggestions for how to manage notifications from Slack/Zulip during the workshop. Several participants requested a companion book for the lecture content and a session on bioinformatics recordkeeping to facilitate reproducibility.

Based on feedback from participants and discussion among the instructors, there are several additional changes that we expect to implement in future online workshops. First, we will provide clearer direction on the purpose of the pod channels and encourage their use by creating small group activities that pods can work on together. These could include providing icebreaker prompts on the first day and group exercises of increasing complexity on subsequent days (e.g., where participants must construct their own series of commands to complete an analysis task). Similarly, to support networking in online events, we are interested in experimenting with pairing individuals for brief (e.g., 5 minutes) one-on-one meetings, either by matching participants based on research interests (as has been reported for other online meetings [7]) or by providing discussion prompts such as “What could we collaborate on?,” which require individuals to give brief “elevator pitches” on their background and interests and then explore how they complement each other.

One change that we made during our first workshop was to create a short refresher video on how to connect to the workshop server. On Day 1 of the workshop, we presented a video that introduced the workshop server and took users through the steps of connecting to the server. We ultimately presented this video multiple times throughout the workshop to help users connect to the server, and, after showing it a few times, it began to feel very repetitive even though it was only 4 minutes long. We decided to edit the video to create a new version with introductory content removed. For technical steps such as this that are repeated throughout the workshop, in the future, we will create an introductory video and then a shorter “refresher video” (that doesn’t, for example, have the instructor introduce themself). This refresher video will only repeat the information that needs to be repeated.

Finally, before workshop registration, we had individuals interested in the CZI/CABANA workshop email to request our purchasing power parity discount. Managing these emails was very labor intensive. In the future, we’ll handle these requests through an online form.

## Conclusions

In response to the COVID-19 pandemic, we have moved the popular QIIME 2 workshop series online. There are undoubtedly drawbacks to holding these types of training events online. Spontaneous conversations often arise at in-person meetings, for example, during dinners or coffee breaks, which lead to new collaborations, employment relationships, and friendships. Teaching and learning can move quickly when a teacher and student can sit together for a few minutes and work on a challenging concept. There are also clear benefits to online events. They are more inclusive, enabling engagement by individuals who are unable or unwilling to travel, and can at least partially remove economic and political barriers to engagement. Travel visas and expensive plane tickets are not needed. Online events can also have a considerably smaller carbon footprint than in-person meetings.

Our approach to hands-on bioinformatics instruction translated well to online delivery. Overall, we did not experience any more technical challenges than we do at similar in-person events. Based on the experiences presented here, we feel that online delivery of bioinformatics education workshops can be effective and empowering. After the pandemic, we expect to continue hosting online workshops.

A recent study on multitasking during remote meetings found that shortening meeting duration, inserting breaks between meetings, and reducing the number of redundant meetings can improve the attendee’s experience by reducing mental fatigue [8]. While our workshop’s structure wasn’t directly modeled after these recommendations, we were happy to find that we fostered several of these best practices. For example, by having clear visibility on the schedule and access to prerecorded lectures, workshop attendees would have the opportunity to skip a presentation if they already had familiarity with the topic or watch a lecture beforehand to accommodate scheduling conflicts.

A benefit of online educational events with prerecorded content is that it prompts the development of video content that can be reused. In January of 2021, we launched the QIIME 2 YouTube channel (https://www.youtube.com/c/qiime2) and are releasing our prerecorded content with accompanying slides under the CC-BY license. To facilitate access to this content, we copyedit auto-generated captions before release, and we ultimately hope to integrate captions in languages other than English. We plan to continue releasing content on this channel with materials from future online workshops, providing new microbiome bioinformatics educational content for researchers and opportunities for QIIME 2 plug-in developers and others to disseminate their work.

## Supporting information

S1 Fig Schedule from our most recent in-person workshop (January 2020). (PDF)

S2 Fig Example “cheat sheet” used to provide server connection instructions and other reference material to the workshop participants. (PDF)

S1 Data Raw data from postworkshop surveys that we summarize in our discussion. This does not include free-form text answers because we did not obtain authorization from workshop participants to share that information.
(XLSX)

S1 Text Preworkshop questionnaire derived from The Carpentries. (PDF)
