# Supplementary material for: Experiences and lessons learned from two virtual, hands-on microbiome bioinformatics workshops
Source: PLoS Comput Biol. 2021 Jun 24;17(6):e1009056. doi: 10.1371/journal.pcbi.1009056 (PMC8224931; doi:10.1371/journal.pcbi.1009056)
Supplement: S1 Fig — (PDF) [file pcbi.1009056.s001.pdf]

Microbiome Bioinformatics with QIIME 2

FAES@NIH workshop (BIOF 089), January 2020

http://bit.ly/2MeDAdh

start of block	**January 8, 2020**	**January 9, 2020**	**January 10, 2020**		
	**Bldg 45; Room E1 & E2**	**Bldg 45; Room E1 & E2**	**Bldg 45; Room E1 & E2**		**Bldg 45; Room H**
7:30	[7:30-8:30] Check-in & registration (Bldg 45; E1 & E2)	[7:30-8:30] Check-in & registration (Bldg 45; E1 & E2)	[7:30-8:30] Check-in & registration (Bldg 45; E1 & E2)		
7:45					
8:00					
8:15					
8:30	Introduction to microbiome, QIIME 2, and plans for the workshop (Greg)	β-diversity metrics, statistics, and visualizations (Aeriel)	Longitudinal analyses (Heather & Bod)	OR	QIIME 2 install clinic (Matt, Evan, Greg, Aeriel)
8:45					
9:00					
9:15					
9:30		Taxonomy assignment and visualizations (Bod)			
9:45	Connecting to the cluster (Matt)				
10:00	Coffee break, open time for discussion with instructors	Coffee break, open time for discussion with instructors	Coffee break, open time for discussion with instructors		
10:15					
10:30	QIIME 2 semantic types and data formats (Matt)	Taxonomy assignment and visualizations (Bod)	Sample classification (Aeriel & Greg)	OR	Round-table discussion: "What do you think of these data?" (Bod, Evan, Heather, Matt)
10:45	Sample metadata and tutorial study background (Matt)				
11:00		Differential abundance testing (Evan)			
11:15	Importing, demux, and denoising (Heather)				
11:30					
11:45					
12:00	Lunch, open time for discussion with instructors	Lunch, open time for discussion with instructors	Lunch, open time for discussion with instructors		
12:15					
12:30					
12:45					
13:00	Importing, demux, and denoising (Heather)	Differential abundance testing (Evan)	Importing data into QIIME 2 (Greg, Bod, Heather, Aeriel)	OR	Beyond the tutorial: advanced techniques and utilities in QIIME 2 (Matt & Evan)
13:15	Phylogenetic reconstruction (Bod)				
13:30		Challenges in Taxonomic classification of microbiome sequence data (Nidhi)			
13:45	Rarefaction (Evan)				
14:00		Open time for discussion with instructors and group question and answer session			
14:15					
14:30	Coffee break, open time for discussion with instructors	Coffee break, open time for discussion with instructors	Coffee break, open time for discussion with instructors		
14:45					
15:00	α-diversity metrics, statistics, and visualizations (Aeriel)	Methods for conducting studies of the human microbiota: Applications for large-scale population-based studies (Emily)	Panel discussion: Career questions (Greg, Aeriel, Bod)	OR	Round-table discussion: "Can I get a second opinion on X?" (Heather, Evan, Matt)
15:15					
15:30					
15:45					
16:00	Open time for discussion with instructors and group question and answer session	Open time for discussion with instructors and group question and answer session	Closing session (Greg)		
16:15					
after hours	Informal & optional mixer	Poster session (1630+; Upstairs atrium)			

Microbiome Bioinformatics with QIIME 2

Water Cooler Chats

FAES@NIH workshop (BIOF 089), January 2020

http://bit.ly/2MeDAdh

start of block		**January 8, 2020**		**January 9, 2020**		**January 10, 2020**	
		**Discussion A**	**Discussion B**	**Discussion A**	**Discussion B**	**Discussion A**	**Discussion B**
10:00	**Coffee break**	TBA	TBA	qiime2R demo and discussion (Evan)	TBA	Vega Visualization Customization (Matt)	IonTorrent (Greg & Evan)
10:15		Chrome & Secure Shell (Evan)	TBA		TBA	TBA	
12:00	**Lunch break**	DNA Subway (Greg)	QIIME 2 install clinic (Matt)	Metagenomic Bias (Bod)	QIIME 2 install clinic (Matt)	TBA	Data formats (Matt)
12:15		TBA				TBA	
12:30		TBA		TBA		TBA	TBA
12:45		Quality Scores (Bod)		Denoising Continued (Heather)		TBA	TBA
14:30	**Coffee break**	Quality Scores cont (Bod)	TBA	TBA	TBA	TBA	TBA
14:45		TBA	TBA	q2studio demo (Matt)	TBA	TBA	TBA
