# Supplementary material for: Experiences and lessons learned from two virtual, hands-on microbiome bioinformatics workshops
Source: PLoS Comput Biol. 2021 Jun 24;17(6):e1009056. doi: 10.1371/journal.pcbi.1009056 (PMC8224931; doi:10.1371/journal.pcbi.1009056)
Supplement: S2 Fig — (PDF) [file pcbi.1009056.s002.pdf]

Supplementary File 2.

CZI-CABANA Microbiome Bioinformatics with QIIME 2 Workshop

October 5 - October 9, 2020

Workshop Tutorial URL: https://docs.qiime2.org/2020.8/tutorials/pd-mice/

This work is licensed under a Creative Commons Attribution-ShareAlike 4.0 International License.

A Greg Caporaso	B migratory-lamprey
C https://workshop-server.qiime2.org/migratory-lamprey	D bbd64ceb85465884a67f

Logging into the Workshop Cluster

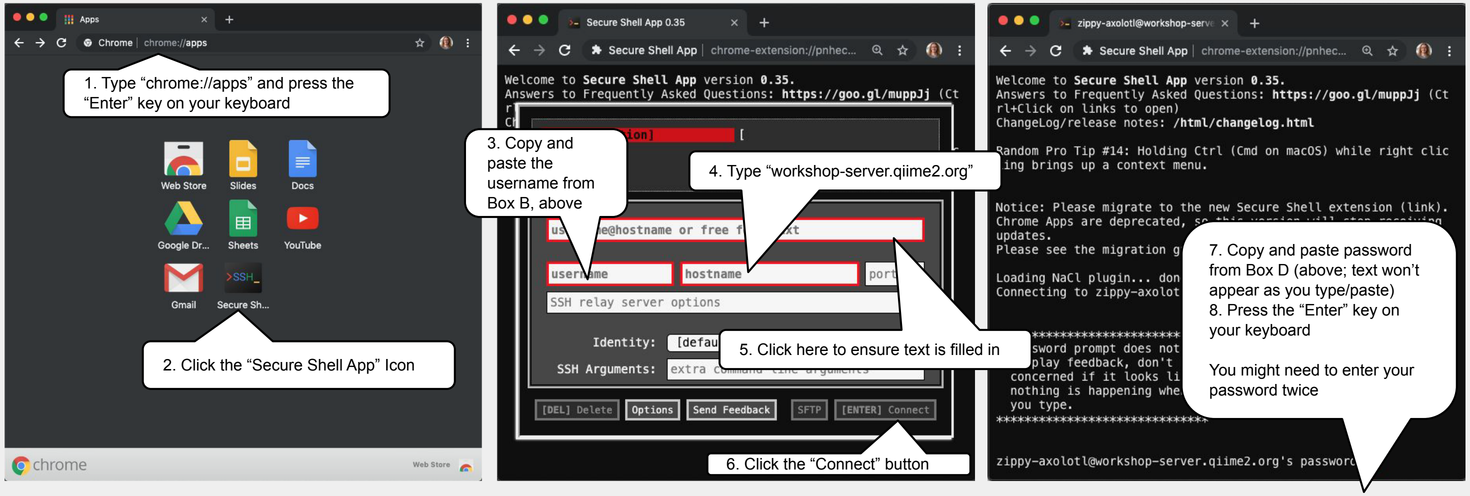

Open Google Chrome and the Secure Shell App → Enter Username and Hostname → Copy-and-paste password

Viewing QIIME 2 Results

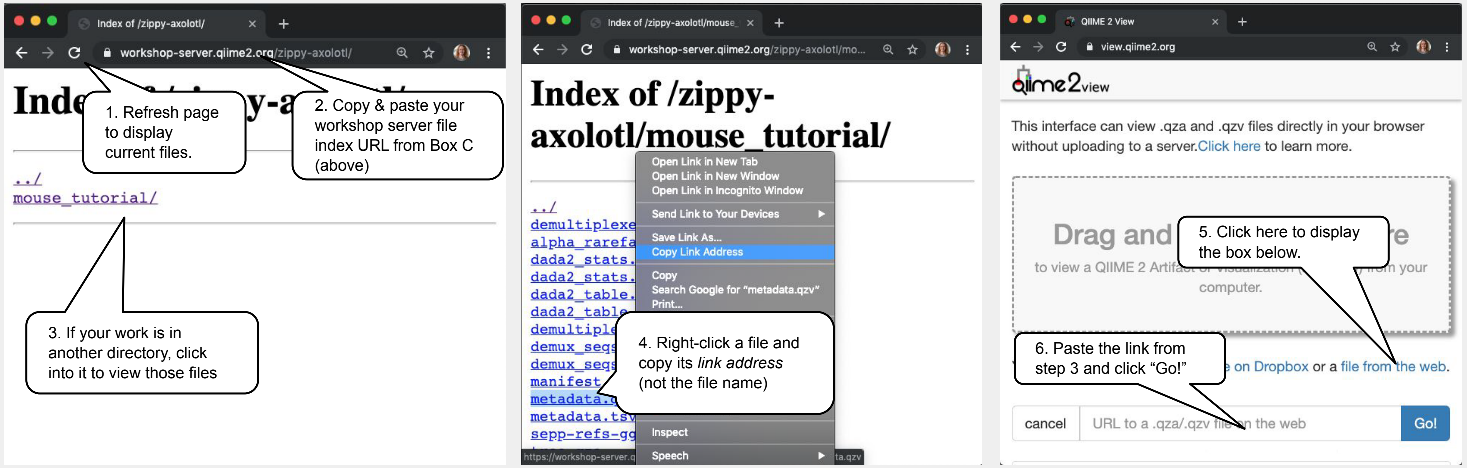

Open Google Chrome and go to your workshop server file index → Copy link to .qzv or .qza file → Paste in at https://view.qiime2.org and click "Go!" button

Important commands

System Navigation

- `ls`
  list contents of current directory
- `cd $PATH`
  change working directory to `$PATH`
- `mkdir $DIR`
  makes a directory named `$DIR`
- `pwd`
  print the location of your current working directory

Moving, renaming, and removing files

- `mv $FILE $DESTINATION`
  moves `$FILE` to `$DESTINATION`
- `cp $FILE $DESTINATION`
  copies `$FILE` to `$DESTINATION`
- `rm $FILE`
  deletes (permanently removes) `$FILE`
- `rm -rf $DIRECTORY`
  deletes (permanently removes) `$DIRECTORY`

Reading and modifying *text* files

- `less $FILENAME`
  displays contents of `$FILENAME`; scroll up/down with arrows; jump to top with `gg`, or bottom with `G`
- `nano $FILENAME`
  opens `$FILENAME` in read/write mode
- `head $FILENAME`
  print the first ten lines of `$FILENAME` to the terminal
- `tail $FILENAME`
  print the last ten lines of `$FILENAME` to the terminal

Other useful commands

- `wget $URL`
  download a file from `$URL`
- `man $COMMAND`
  learn about a command

QIIME 2 Links

View: https://view.qiime2.org
Forum: https://forum.qiime2.org
Documentation: https://docs.qiime2.org/2020.8/
Community plugins: https://library.qiime2.org
"Core" plugins: https://docs.qiime2.org/2020.8/plugins/
