# Supplementary material for: Experiences and lessons learned from two virtual, hands-on microbiome bioinformatics workshops
Source: PLoS Comput Biol. 2021 Jun 24;17(6):e1009056. doi: 10.1371/journal.pcbi.1009056 (PMC8224931; doi:10.1371/journal.pcbi.1009056)
Supplement: S1 Text — (PDF) [file pcbi.1009056.s004.pdf]

CZI-CABANA Microbiome Bioinformatics with QIIME 2 Workshop

5 October - 9 October 2020

* Required

1. Email *

____

2. Please indicate your relevant fields or disciplines. *

Check all that apply.

- [ ] Agricultural or Environmental Sciences
- [ ] Biomedical or Human Health Sciences
- [ ] Chemistry
- [ ] Computer Science or Electrical Engineering
- [ ] Education
- [ ] Bioinformatics
- [ ] High Performance Computing
- [ ] Mathematics or Statistics
- [ ] Medicine
- [ ] Microbiology
- [ ] Organismal Biology (Ecology, Botany, Zoology, etc)
- [ ] Planetary Sciences (Geology, Climatology, Oceanography, etc.)
- [ ] Psychology or Neuroscience
- [ ] Space Sciences
- [ ] Veterinary Sciences or Veterinary Medicine

Other: [ ] _______________________________

3. What is your current occupation/career stage? *

Mark only one oval.

- ( ) Administrative Staff
- ( ) Analyst
- ( ) Commercial Software Developer
- ( ) Faculty
- ( ) Government Employee
- ( ) Graduate Student
- ( ) Industry Employee
- ( ) Librarian or Archivist
- ( ) Management
- ( ) Medical, Nursing, or Other Medical Professional Student
- ( ) Physician or Other Medical Professional
- ( ) Postdoctoral Researcher
- ( ) Research Staff (including Researcher Programmer)
- ( ) Retired or Not Employed
- ( ) Support Staff (including Technical Support)
- ( ) Undergraduate Student
- ( ) Other: ____________________

4. What operating system is on the computer you are using at the QIIME 2 workshop? *

Check all that apply.

- [ ] macOS
- [ ] Linux
- [ ] Windows
- [ ] Not sure

Other: [ ] ____________________

5. How often do you currently use specialized software with a point-and-click graphical user interface (e.g., for statistical analysis: SPSS, SAS, ...; for Geospatial analysis: ArcGIS, QGIS, ... ; for Genomics analysis: Geneious, ...)? *

Mark only one oval.

- Never
- Less than once per year
- Several times per year
- Monthly
- Weekly
- Daily

6. How often do you currently use programming languages (R, Python, etc.)? *

Mark only one oval.

- Never
- Less than once per year
- Several times per year
- Monthly
- Weekly
- Daily

7. How often do you currently use databases (SQL, Access, etc.)? *

Mark only one oval.

- Never
- Less than once per year
- Several times per year
- Monthly
- Weekly
- Daily

8. How often do you currently use version control software (Git, Subversion (SVN), Mercurial, etc.)? *

Mark only one oval.

- Never
- Less than once per year
- Several times per year
- Monthly
- Weekly
- Daily

9. How often do you currently use a command shell (usually accessed through Terminal on macOS or PowerShell on Windows)? *

Mark only one oval.

- Never
- Less than once per year
- Several times per year
- Monthly
- Weekly
- Daily

10. How often do you currently use QIIME 2? *

Mark only one oval.

- Never
- Less than once per year
- Several times per year
- Monthly
- Weekly
- Daily

11. How often do you currently use QIIME 1? *

Mark only one oval.

- Never
- Less than once per year
- Several times per year
- Monthly
- Weekly
- Daily

12. Please rate your level of satisfaction with your current data management and analysis workflow (i.e. how you collect, organize, store and analyze your data). *

Mark only one oval.

- Very unsatisfied
- Unsatisfied
- Neutral
- Satisfied
- Very satisfied
- Not sure
- Not applicable
- Never thought about this

13. Why are you participating in this workshop? *

Mark only one oval.

- To learn new skills.
- To refresh or review my skills.
- To learn skills that I can apply to my current work.
- To learn skills that I can apply to my work in the future.
- To learn skills that will help me get a job or a promotion.
- As a requirement for my program or current position.
- Other: ______________________

14. How did you find out about this workshop? *

Mark only one oval.

- ( ) Received an email or saw a flyer about the workshop
- ( ) Read about the workshop in a newsletter or on university website
- ( ) Saw the workshop listed on forum.qiime2.org or workshops.qiime2.org
- ( ) My advisor or supervisor told me about the workshop
- ( ) A friend or colleague told me about the workshop
- ( ) Saw the workshop advertised on social media (Twitter, Facebook, etc.)
- ( ) Heard about the workshop at a conference, meeting, or seminar
- ( ) Heard about the workshop from a funding organization or program officer
- ( ) Other: ______________________

15. Please rate your level of agreement with this statements: Having access to the original, raw data is important to be able to repeat an analysis. *

Mark only one oval.

	1	2	3	4	5	
Strongly Disagree	( )	( )	( )	( )	( )	Strongly Agree

16. Please rate your level of agreement with this statement: I can write a small program, script, or macro to address a problem in my own work. *

Mark only one oval.

	1	2	3	4	5	
Strongly disagree	( )	( )	( )	( )	( )	Strongly agree

17. Please rate your level of agreement with this statement: I know how to search for answers to my technical questions online. *

Mark only one oval.

	1	2	3	4	5	
Strongly disagree	◯	◯	◯	◯	◯	Strongly agree

18. Please rate your level of agreement with this statement: I am confident in my ability to make use of programming software to work with data. *

Mark only one oval.

	1	2	3	4	5	
Strongly disagree	◯	◯	◯	◯	◯	Strongly agree

19. Please rate your level of agreement with this statement: Using a programming language (like R or Python) can make my analyses easier to reproduce. *

Mark only one oval.

	1	2	3	4	5	
Strongly disagree	◯	◯	◯	◯	◯	Strongly agree

20. Please rate your level of agreement with this statement: Using a programming language (like R or Python) can make me more efficient at working with data. *

Mark only one oval.

	1	2	3	4	5	
Strongly disagree	◯	◯	◯	◯	◯	Strongly agree

21. Please share what you most hope to learn from participating in this workshop.

The content in this survey is derived from the The Carpentries, where it is made available under the CC-BY License. We have adapted this content for this survey. The original materials are available at https://github.com/carpentries/assessment.

This content is neither created nor endorsed by Google.

Google Forms
